# The resting coronal and sagittal stance position of the torso in adolescents with and without spinal deformity

**DOI:** 10.1038/s41598-021-81818-z

**Published:** 2021-01-27

**Authors:** Adrian Gardner, James Archer, Fiona Berryman, Paul Pynsent

**Affiliations:** 1 University of Birmingham, Birmingham, UK; 2 The Royal Orthopaedic Hospital NHS Foundation Trust, Birmingham, UK

**Keywords:** Musculoskeletal system, Paediatric research

## Abstract

The purpose of this work is to identify the resting stance of the torso, defined as the position of the C7 vertebral body relative to the sacrum in a ‘birds eye view’, as the coronal and sagittal offset, in those without spinal deformity, those with pre and post-operative AIS, and those with Scheuermann's kyphosis (SK). Using ISIS2 surface topography, the coronal and sagittal offset were measured in a prospective manner in all groups. With bivariate ellipses, a mean and 95% confidence ellipse of the data was developed. Statistical analyses was performed to examine the distribution of the data from the groups. A graphical representation of the data was developed. There were 829 without spinal deformity, 289 in both the pre and post-operative with AIS and 59 with SK. The results showed that the mean coronal offset for all groups was between 2 and 6 mm and the sagittal offset was 12 and 26 mm. Statistically significance was seen for both measures between the non-scoliotic and both AIS groups, along with the pre-operative AIS coronal offset and post-operative AIS sagittal offset and the SK measures. However, all mean values were within the 95% confidence ellipse for all of the groups. Regardless of the size or type of spinal deformity, the position of the C7 vertebral body and sacrum remain within the 95% confidence ellipse of that seen in those without spinal deformity. This work defines the Minimally Clinically Important Difference for all of the groups.

## Introduction

Adolescent spinal deformity, resulting from either idiopathic scoliosis (AIS) or Scheuermann’s kyphosis (SK), leads to a deformity that develops in the spine and torso during the adolescent growth spurt^1, 2^. This change in shape can alter the relationship of the head to the pelvis in 3D space^3^. Consequently, the assessment of an individual with spinal deformity requires an assessment of the position of the head relative to the sacrum, which is described as coronal and sagittal offset^4^. The Scoliosis Research Society defines coronal offset is defined as the horizontal distance in the coronal plane between a vertical line dropped from the C7 vertebral body and a vertical line through the centre of the sacrum^5^. Sagittal offset is defined as the horizontal distance between the C7 vertebral body and the most posterior superior corner of the sacrum in the sagittal plane^5^. The parameters of coronal and sagittal offset can be assessed both clinically, using surface topography and from radiographs.

The importance of the sagittal and coronal offsets relates to the cone of economy which was first described by Professor Dubousset in 1994^6^. It is an anatomical and physiological concept that describes the range of body positions within which upright stance can be maintained. The cone of economy is an upside down cone, with the feet at the apex of the cone. As the centre of mass of the body moves towards the edges of the cone, the muscular activity required to maintain upright stance increases. It is biomechanically most energy efficient to have as little coronal and sagittal offset as possible^7^. Of note, the position of the body in the cone of economy is comprised of the positions of both the axial and appendicular skeletons. Spinal surgeons act to affect the position of the axial skeleton, in the knowledge that this can cause compensatory changes in the appendicular skeleton that occur as a result of spinal surgery. An equivalent of the cone of economy for the spine would be a cone with the apex inferiorly at the sacrum rather than the feet. One of the goals of surgery on AIS patients is therefore to reduce these offsets, together with all other components of spinal deformity, towards the normal levels expected in non-scoliotic individuals^8^.

Assessments of the range of normality in the measures of coronal and sagittal offset have been reported in the literature using a number of different measurement techniques^9–13^. The literature suggests that a coronal offset of 0 mm ± 28 mm (mean ± 95% CI) and a sagittal offset of between 90 mm of negative offset (C7 vertebral body or vertebra prominens (VP) posterior to the sacrum) and 61 mm of positive offset (C7 or VP anterior to the sacrum) (± 95% CI) would be accepted as representing a non-pathologic state^9–13^. However, these values are one dimensional, being reported separately from each other. It would be more useful to be able to assess the coronal and sagittal offsets together. Although not illustrating the dynamic positions of the body whilst being able to maintain upright stance as in the cone of economy, the description of coronal and sagittal offsets would give a description of the variability in a resting upright position for a population. Plotting the coronal and sagittal offsets measured at the same time from a bird’s eye viewpoint and using data ellipses^14, 15^ allows an analysis of this to be achieved. Knowledge of the normal range of the variability of coronal and sagittal offsets for those with and without spinal deformity would be very useful in understanding the effects of both AIS and SK on the shape of the spine in reference to normative values.

Although coronal and sagittal offsets can be measured from radiographs, it would be unethical to expose non-scoliotic individuals to unnecessary radiation. Surface topography, a technique that is free of ionising radiation, is ideally suited for investigating the shape of those who do not otherwise need a radiograph^16^ and also gives data across different populations that are directly comparable.

The purpose of this study is to use the ISIS2 (Integrated Shape Imaging System 2)^17^ surface topography to measure and compare the coronal and sagittal offsets in a number of adolescents without spinal deformity, comparing that to those with SK or AIS, the latter both pre-operatively and post-operatively.

## Methods

The study design is a comparison of measured parameters of spinal position in four different cohorts representing those without spinal deformity and comparing to those with AIS, both pre and post-operatively, and those with SK. All participants were measured using the Integrated Shape Imaging System 2 (ISIS2)^17^. ISIS2 is a surface topography system that has been used for research in spinal deformity previously^16^.

Those without scoliosis consisted of a number of children from a local school who were part of a prospective longitudinal study undergoing annual serial imaging using ISIS2 over a period of seven years between 2011 and 2017 inclusive. This was NRES (National Research Ethics Service, UK) and HRA (Health Research Authority, UK) approved and all methods were carried out in accordance with relevant guidelines and regulations with all experimental protocols approved by NRES committee West Midlands—South Birmingham (11/H1207/10). Informed consent was obtained from all subjects or, if subjects are under 18, from a parent and/or legal guardian. None of these children had any form of spinal deformity to clinical examination. Any spine or torso deformity and / or surgery was an exclusion criteria from this group.

Those with AIS consisted of a number of children with AIS who had undergone deformity correction surgery where ISIS2 surface topography images were taken as part of routine care, which were then reviewed at a later date. The inclusion criteria was the diagnosis of AIS which was confirmed using radiographs and exclusion the inability to stand still enough to capture the topography image. The most proximal level of fusion was T2 in the cohort. All individuals in this group underwent imaging with ISIS2 before and after the surgery.

The inclusion criteria for those with SK were kyphosis without a significant scoliotic component (coronal deformity < 20°), and that had not undergone surgery, again with ISIS2 images taken as part of routine care. The diagnosis was confirmed using the radiographs and exclusion the inability to stand still enough to capture the topography image. This review of ISIS2 images was also an NRES and HRA approved study (NRES Committee East Midlands—Northampton 15/EM/0283).

**Figure 1:**
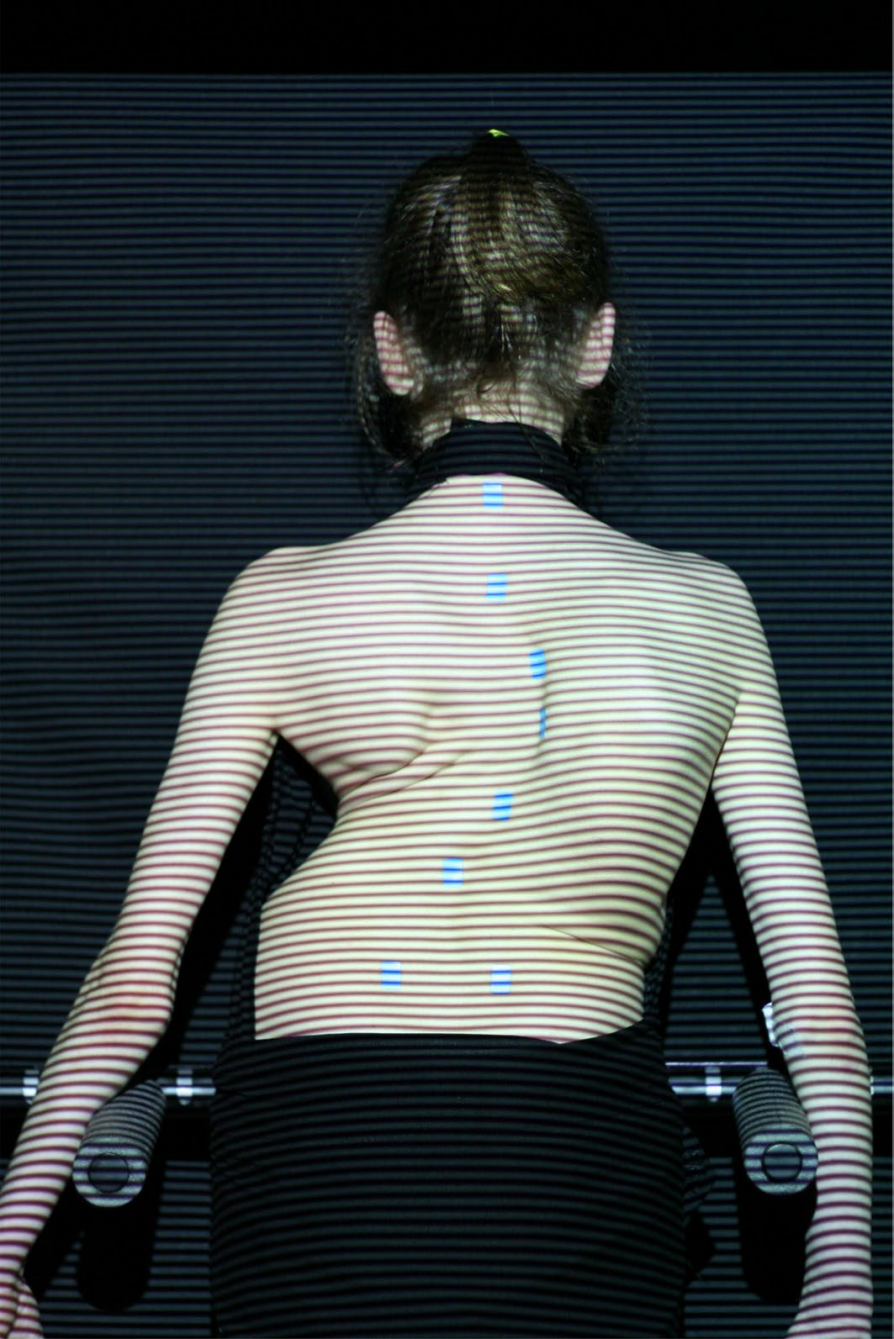
The ISIS2 clinical image with stickers placed over the spine, with the most superior sticker over the VP and the most inferior stickers over the PSIS.

**Figure 2:**
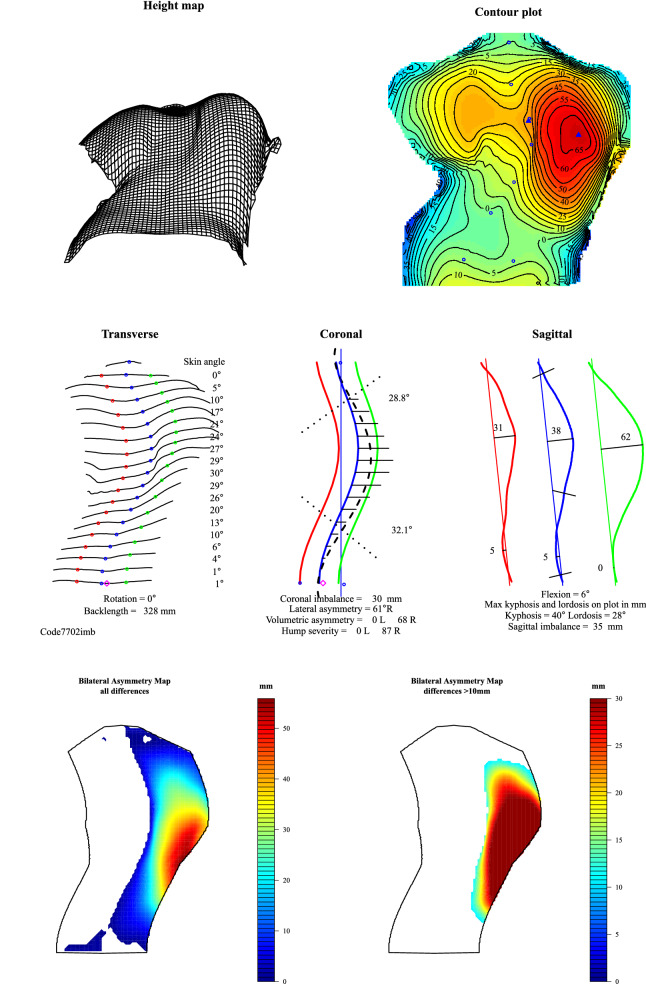
The ISIS2 analysis, describing a number of parameters of spinal and torso shape.

**Figure 3:**
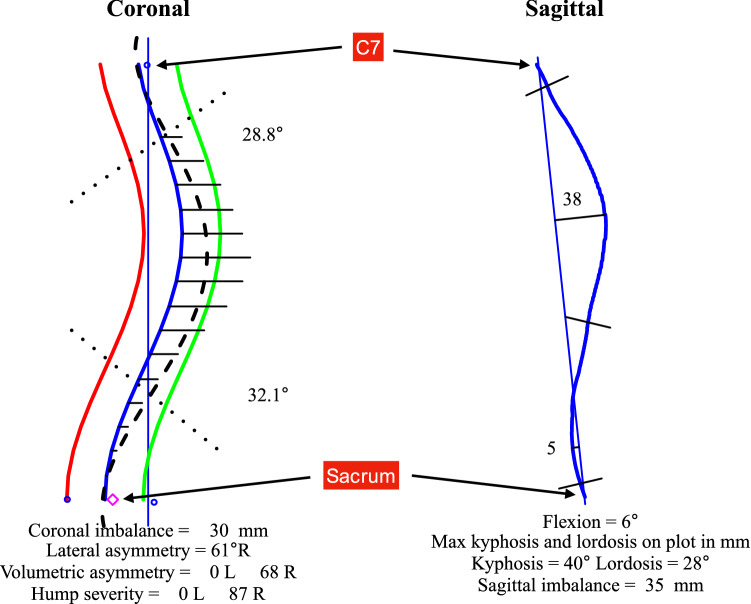
A close up of the key features of the ISIS2 analysis showing both the coronal and sagittal shape of the spine where the position of C7 and the sacrum are highlighted. The measures of coronal and sagittal imbalance are automatically recorded as shown.

When imaging with the ISIS2 system, the individual stands comfortably in their ‘resting’ stance, with the feet hip width apart and arms by the side, slightly away from the torso (Figs. 1, 2 and 3). There is an abdominal bar in front of the individual which allows a reference point to help to prevent sway, but the individual does not lean against it. ISIS2 measures and reports the 3D position of the vertebral body of C7, which is calculated from the surface position of the vertebra prominens (VP) using established methodology^17^. By measuring the 3D position of C7 relative to the 3D position of the sacrum, calculated from the positions of the palpated posterior superior iliac crests (PSIS), the coronal and the sagittal offset can be reported. By definition in this study, the coronal and sagittal offset would measure 0 mm when the position of the C7 was directly superior to that of the sacrum, as if a plumb line had been dropped from C7 to the sacrum. Thus coronal offset is when there is a difference in position between the position of the C7 and sacrum in the coronal plane and sagittal offset, when there is a difference in position between the position of C7 and sacrum in the sagittal plane. For coronal offset, a positive number is when C7 is to the right of the sacrum, a negative number when C7 is to the left of the sacrum. For sagittal offset, a positive number is when C7 is anterior to the sacrum and a negative when posterior to the sacrum.

All analysis of the data was performed using R^18^ with the data ellipse methodology described previously by our group^16^. The position of C7 can be seen from a bird’s eye position (looking down from above) relative to the sacrum using anatomical terms for orientation. Using bivariate data ellipses^15^ with the R car package^14^, the mean position for the cohorts, non-scoliotic, pre-operative and post-operative AIS and SK, were identified along with the 95% confidence ellipse of the mean. Any statistically significant differences between the positions of the mean points were established using Students t tests, paired for the pre-operative to post-operative AIS data and unpaired for comparisons of the non-scoliotic to the pre-operative, post-operative AIS and SK data. Analysis was performed both for Lenke coronal subtypes^19^ and between males and females for both coronal and sagittal offset in all groups to examine whether sex was a statistically significant factor. The size of all spinal deformities were measured with the Cobb angle^20^. Statistical significance was pre-defined as p < 0.05.

## Results

In those without scoliosis, there were 829 individual ISIS2 images from 117 males and 77 females. In those with AIS, there were 289 individuals providing both pre-operative and post-operative information consisting of 39 males and 250 females. In those with SK, there were 59 individuals, 22 females and 37 males. The demographic information from the three cohorts is found in Table 1 including the time between the ISIS2 image and surgery, both pre and post-operative, for this with AIS.

**Table 1:** The demographic details of the non-scoliotic, scoliotic and SK groups.

	Mean age (years)	SD (years)	Range (years)	Males	Females	Number of individual images	Size of scoliotic/kyphotic curve, mean (SD) (°)	Time between image and surgery, median (IQR) (days)
Non-scoliotic	12.9	1.9	9.2–17.9	117	79	829	NA	NA
Pre-operative AIS	13.9 (at pre-operative image)	1.5	9.9–17.9	39	250	289	58 (19)	361 (391)
Post-operative AIS	15.7 (at post-operative image)	1.6	12.0–19.0	39	250	289	21 (11)	187 (249)
SK	15.8	1.5	13.2–19.0	37	22	59	68 (7)	NA

Table 2 records the mean, standard deviation and range of the coronal and sagittal offset for each of the groups. Table 3 records the statistical analysis of the differences in the mean values between the groups. There were statistically significant differences between the data for all of the comparisons between the non-scoliotic and the pre-operative AIS group, the non-scoliotic and post-operative AIS groups and between the pre-operative and post-operative AIS group. There were also statistically significant differences between the pre-operative AIS group and the SK groups for coronal imbalance and the post-operative AIS group and the SK group for sagittal imbalance.

**Table 2:** The mean, standard deviation and range of the coronal and sagittal offsets in the non-scoliotic, scoliotic and SK groups. Please note that the images in the pre-operative and post-operative AIS groups are paired.

	Coronal offset (mm)	Sagittal offset (mm)
Non-scoliotic	6 ( 9, − 22 to 33)	26 (19, − 39 to 83)
Pre-operative AIS	2 (20, − 56 to 52)	23 (22, − 47 to 94)
Post-operative AIS	4 (16, − 35 to 55)	12 (24, − 43 to 111)
SK	6 (12, − 27 to 38)	20 (26, − 62 to 67)

**Table 3:** The statistical significance between the measures of coronal and sagittal offset in the non-scoliotic, scoliotic and SK groups.

	Coronal offset	Sagittal offset
Non-scoliotic versus pre-operative AIS	< 0.001	0.013
Non-scoliotic versus post-operative AIS	0.016	< 0.001
Pre-operative versus post-operative AIS	< 0.001	< 0.001
Non-scoliotic versus SK	0.848	0.096
Pre-operative AIS versus SK	0.048	0.527
Post-operative AIS versus SK	0.536	0.035

The AIS group was also analysed with the data subdivided in to the coronal subtypes of the Lenke classification^19^. The data was comprised of Lenke 1 and Lenke 5 curves only. There was a statistically significant difference seen (p < 0.0001) between the coronal offset of the Lenke 5 curves when compared to all of the data and also the Lenke 1 curves with an absolute difference of 20 mm. However, the mean values all were within the 95% confidence intervals of each other.

Statistically significant differences were seen (p < 0.05) between the sexes for only sagittal imbalance for those without scoliosis and for both the pre-operative and post-operative AIS groups but not for the SK group. There were no statistically significant differences for coronal imbalance in any of the groups. Examining the size of the mean differences in the parameters between the sexes for each of the groups showed that the maximum difference was 6 mm for coronal offset and 11 mm for sagittal offset.

**Figure 4:**
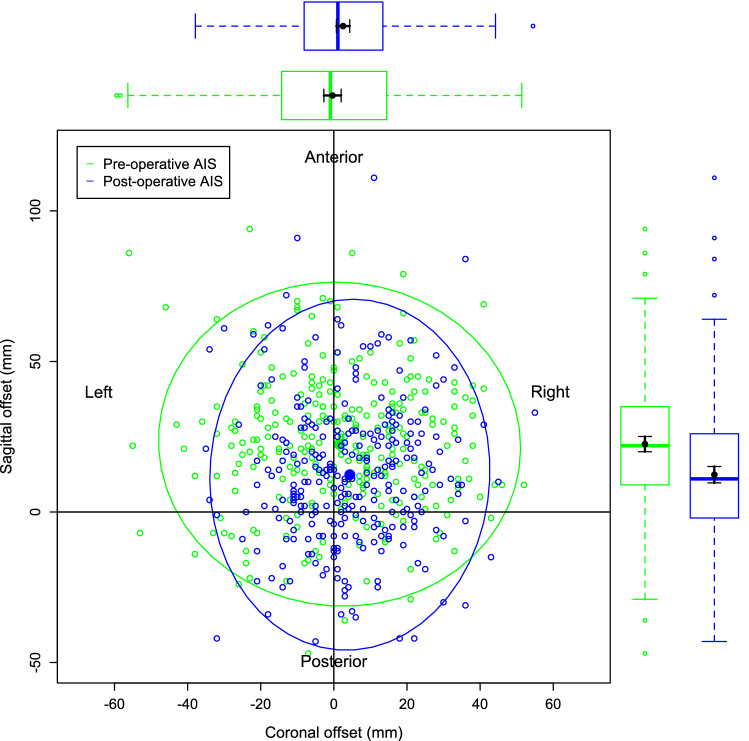
The individual data points, mean values and 95% confidence ellipses for the pre and post-operative AIS groups. The box and whisker plots demonstrate the data of the equivalent parameter in that group. The plot shows the data from a ‘bird’s eye’ position with anatomic descriptors of ‘Anterior’, ‘Posterior’, ‘Left’ and ‘Right’ as marked.

**Figure 5:**
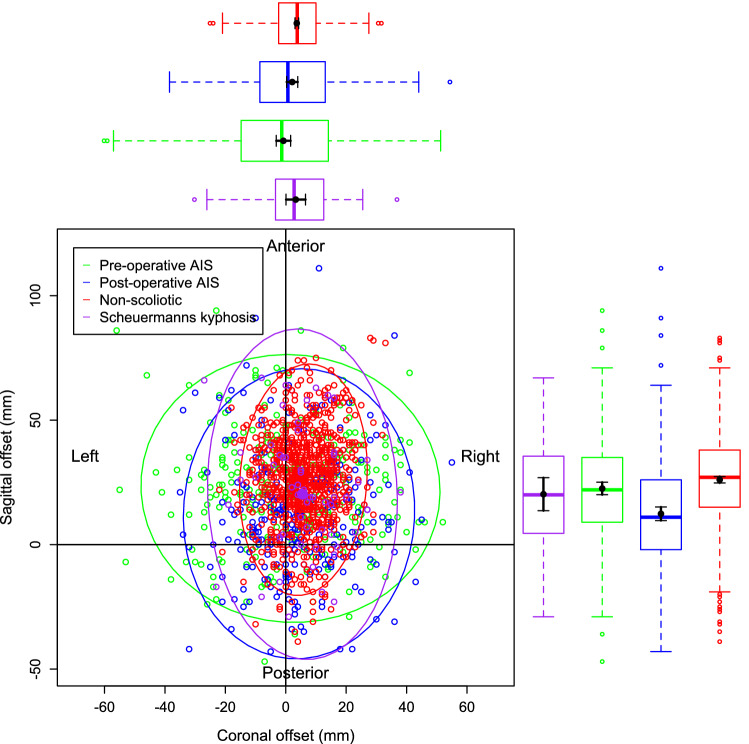
The individual data points, mean values and 95% confidence ellipses for the non-scoliotic (red), pre-operative AIS (green), post-operative AIS (blue) and SK (purple) groups demonstrating all of the data as open circles, the mean values as closed circles and the 95% confidence ellipses for that group.

**Figure 6:**
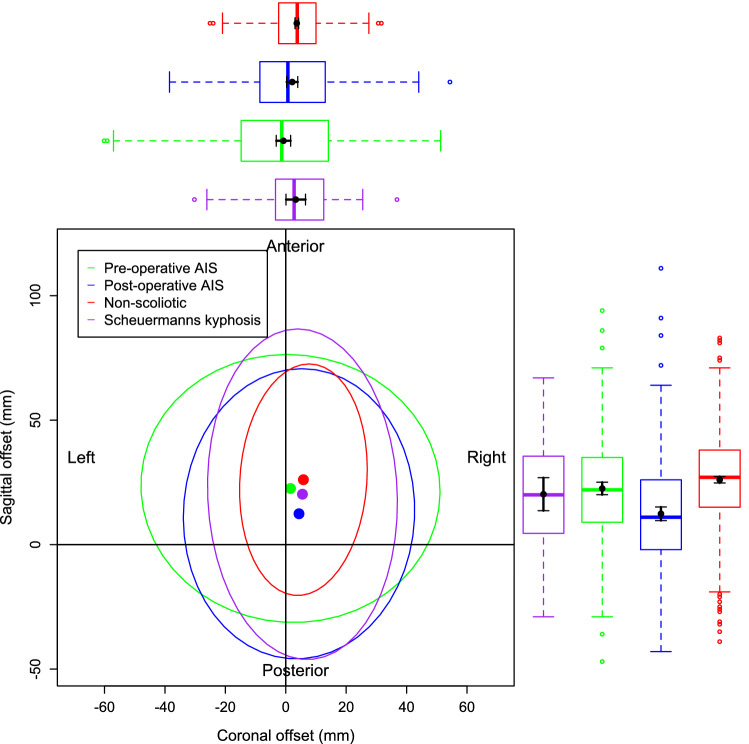
The mean values and 95% confidence ellipses for the non-scoliotic (red), pre-operative AIS (green), post-operative AIS (blue) and SK (purple) groups. This plot only shows the mean values and 95% confidence ellipses for clarity.

Figure 4 is a visual representation of the bivariate nature of the data for the pre-operative and post-operative AIS groups only. This is to illustrate the relationship between the individual data points, the mean of those data points and the 95% confidence ellipse of the data. Figure 5 demonstrates the data and the 95% confidence ellipses for the non-scoliotic, pre and post-operative AIS and the SK groups. Figure 6 demonstrates the mean with only the 95% confidence ellipses for all of the groups. The individual data points are removed in Fig. 6 for clarity as the data points within the data ellipses obscures relevant details. In all of the figures, the non-scoliotic data is in red, the pre-operative AIS data in green, the post-operative AIS data in blue and the SK data is in purple. Box and whisker plots are seen in the same colors on both the x and y axes with both the mean and median values shown. For clarification, Figs. 4, 5 and 6 are plotted from a bird’s eye position, with anterior, posterior, left and right as marked. If there was no difference between the positions of C7 and sacrum in terms of coronal and sagittal offsets, then the data point would be at the origin.

## Discussion

**Figure 7:**
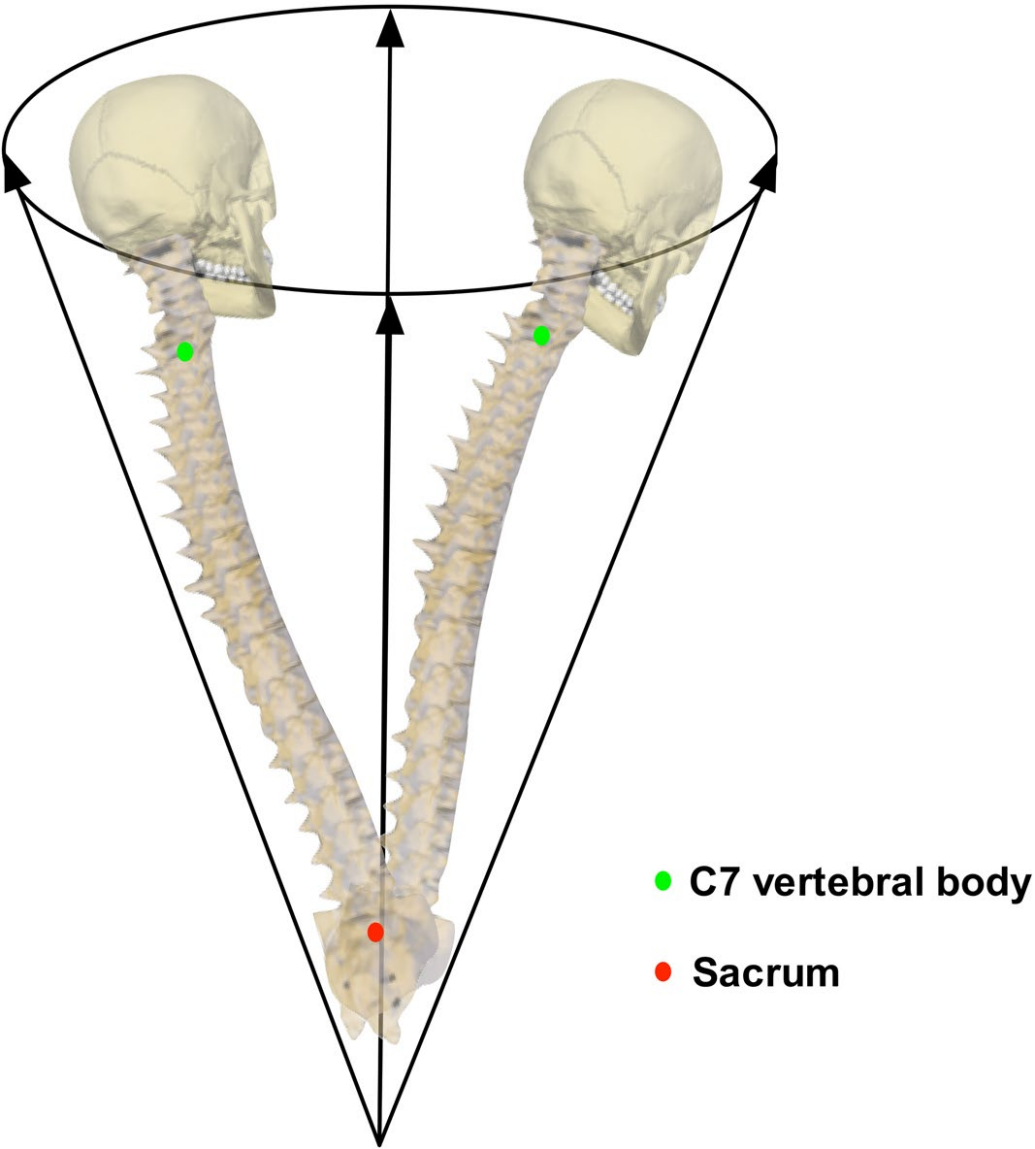
A diagrammatic representation of the ‘variability of the coronal and sagittal offset’ as described in this paper.

As with all measures of the position of the body there is a range of values that represent normality. Being able to establish the range of normality is important as this leads on to the development of the Minimal Clinically Important Difference (MCID)^21^. The MCID represents the minimum amount of change in a parameter that is deemed of sufficient size to be of clinical import to either the patient or clinician^21^. Thus it is possible for observations to be statistically significant but clinically insignificant. The measure of the mean and 95% confidence ellipse for the coronal and sagittal imbalance in the non-scoliotic cohort establishes normative values for future work. The 95% confidence ellipse can be viewed as the MCID for both the coronal and sagittal offset between C7 and the sacrum (Fig. 7) where a cone is described of the spine only with the apex of the cone at the sacrum, in distinction to the cone of economy described by Dubousset^6^ with the apex at the feet. The MCID from the confidence ellipse for coronal offset from this work confirms the previously published amount of ± 20 mm^11–13^. The sagittal offset is larger than the values previously quoted the literature^10^ who, using a technique of measurements from a plumb line dropped behind the subject, recorded the sagittal balance (a difference of horizontal distance from the plumb line between that measured at the C7 and S2 vertebral levels) as 19.3 ± 17 mm. More recently however, Clement^9^ recorded the sagittal offset in a group of adolescents, aged between 10 and 18 years of age, as between 90 mm of negative offset with the VP posteriorly positioned relative to the sacrum and 61 mm of positive offset with the VP positioned anteriorly to the sacrum.

Knowledge of the MCID for the coronal and sagittal offset in a non-scoliotic population is important as it allows comparisons to be made with both AIS and SK populations. The work presented here includes those with AIS made up of all curve types, although analysis of Lenke coronal subtypes^19^ has been performed. As the technique used in the identification and measurement of the coronal and sagittal offset is the same for all, a direct comparison can be made. It is not possible to perform a similar study with radiographs due to the ethical issues of irradiating normal children. Combining a surface topography technique for the non-scoliotics and a radiograph for those with AIS or SK would lead to measurement error as the techniques for obtaining the data would be different. The presence of a coronal curve in the pre-operative AIS group demonstrates an alteration in the position of the mean value of both the coronal and sagittal offset when compared to the non-scoliotic group. This is statistically significant but smaller than the established MCID from the non-scoliotic group. Scoliosis surgery reduces the size of the scoliotic curve and alters the amount of kyphosis and lordosis seen in the sagittal profile^22^. The mean position of the coronal and sagittal offsets for the post-operative AIS group is significantly different statistically to that of the non-scoliotic and pre-operative scoliotic groups. However, the size of the differences is again less than the MCID.

With regards to the SK group, there is a significant difference between the coronal imbalance of the pre-operative AIS and SK and in sagittal imbalance between the post-operative AIS and SK. Again, whilst statistically significant, the differences are smaller than the MCID described for all of the comparisons.

The conclusion drawn here is that, despite the change in the shape of the spine between C7 and the sacrum caused by either the AIS or the SK, the body can compensate for this and maintain the position of C7 within the normal range of those without spinal deformity. Scoliosis surgery changes the shape of the spine with instrumentation and fusion. The results presented here show that, following the surgery, the mean position of C7, in terms of coronal and sagittal offset, is maintained within the MCID value established from those without scoliosis. This demonstrates again the ability of the body to compensate for the changes in the shape that comes with scoliosis surgery to allow maintenance of a biomechanically efficient posture.

The work reported in this paper does not establish a cone of economy as this work does not indicate the position of C7, relative to the sacrum, at the extremes of body position at which it is just possible to maintain upright stance, and is not a dynamic concept. However, this work does establish resting coronal and sagittal stance positions of the torso reflecting the position taken by the body in both the coronal and sagittal plane in the resting position. As might be expected, scoliosis leads to a larger ellipse, both pre and post-operatively, than that seen in those without scoliosis, and this is recognised by the assessment of coronal and sagittal offset made in AIS^3^. More importantly, normative values are established for the future. The work presented here builds on the Da Vinci plots that have been used to demonstrate the 3D shape of the spine in AIS^23^.

The strengths of this work are the use of the same ISIS2 surface topography methods in all groups. This removes the possibility of errors occurring through the use of different techniques in the different populations. The use of surface topography also removes any iatrogenic effects on sagittal offset that could occur with positioning of the arms to allow the imaging of the proximal thoracic spine throughout the bulk of the shoulder girdle^24^, as with ISIS2, the arms hang down by the side of the subject. It must however be recognised that the use of surface topography as the measurement tool in this study means that the results are not directly comparable to studies where radiographs have been used to obtain similar parameters. This work could be expanded by using a system that allows the identification of the centre of gravity of the body, similar to that used by Haddas et al.^25^, allowing a dynamic assessment.

In conclusion, this paper demonstrates the mean and 95% confidence ellipses of the combined coronal and sagittal offset in a non-scoliotic group, alongside matched pre-operative and post-operative AIS groups and an SK group. This work establishes the MCID for future work and demonstrates the ability of the spine to compensate, maintaining C7 over the sacrum despite changes in shape and subsequent fusion surgery of the spine between these anatomical points.
